# Factors affecting the chemical efficacy of 2% sodium hypochlorite against oral steady‐state dual‐species biofilms: Exposure time and volume application

**DOI:** 10.1111/iej.13102

**Published:** 2019-03-13

**Authors:** X. Petridis, F. H. Busanello, M. V. R. So, R. J. B. Dijkstra, P. K. Sharma, L. W. M. van der Sluis

**Affiliations:** ^1^ Department of Conservative Dentistry Center for Dentistry and Oral Hygiene University Medical Center Groningen University of Groningen Groningen The Netherlands; ^2^ Conservative Dentistry Department School of Dentistry Federal University of Rio Grande do Sul Porto Alegre Rio Grande do Sul Brazil; ^3^ Department of Biomedical Engineering University Medical Center Groningen University of Groningen Groningen The Netherlands

**Keywords:** 2% NaOCl, Biofilm, optical coherence tomography, removal, time, volume

## Abstract

**Aim:**

To study the influence of time and volume of 2% sodium hypochlorite (NaOCl) on biofilm removal and to investigate the changes induced on the biofilm architecture. Steady‐state, dual‐species biofilms of standardized thickness and a realistic contact surface area between biofilms and NaOCl were used.

**Methodology:**

*Streptococcus oralis* J22 and *Actinomyces naeslundii* T14V‐J1 biofilms were grown on saliva‐coated hydroxyapatite discs within sample holders in the Constant Depth Film Fermenter (CDFF) for 96 h. Two per cent NaOCl was statically applied for three different time intervals (60, 120 and 300 s) and in two different volumes (20 and 40 μL) over the biofilm samples. The diffusion‐driven effects of time and volume on biofilm disruption and dissolution were assessed with Optical Coherence Tomography (OCT). Structural changes of the biofilms treated with 2% NaOCl were studied with Confocal Laser Scanning Microscopy (CLSM) and Low Load Compression Testing (LLCT). A two‐way analysis of variance (2‐way anova) was performed, enabling the effect of each independent variable as well as their interaction on the outcome measures.

**Results:**

Optical coherence tomography revealed that by increasing the exposure time and volume of 2% NaOCl, both biofilm disruption and dissolution significantly increased. Analysis of the interaction between the two independent variables revealed that by increasing the volume of 2% NaOCl, significant biofilm dissolution could be achieved in less time. Examination of the architecture of the remaining biofilms corroborated the EPS‐lytic action of 2% NaOCl, especially when greater volumes were applied. The viscoelastic analysis of the 2% NaOCl‐treated biofilms revealed that the preceding application of higher volumes could impact their subsequent removal.

**Conclusions:**

Time and volume of 2% NaOCl application should be taken into account for maximizing the anti‐biofilm efficiency of the irrigant and devising targeted disinfecting regimes against remaining biofilms.

## Introduction

The oxidizing properties and reactivity of sodium hypochlorite (NaOCl) with organic matter account for its antibacterial and dissolving effect (McDonnell & Russell 1999, Estrela *et al*. 2002), hence rendering it a suitable anti‐biofilm root canal irrigant (Chávez de Paz *et al*. 2010, Tawakoli *et al*. 2015). The factors that affect the reactivity and subsequent dissolving capacity (chemical efficacy) of NaOCl have been primarily investigated on pulp tissue samples, artificial organic films and dentine (Rosenfeld *et al*. 1978, Moorer & Wesselink 1982, Zehnder *et al*. 2002, Sirtes *et al*. 2005, Huang *et al*. 2008, Macedo *et al*. 2010, 2014a,b, Jungbluth *et al*. 2011). This makes extrapolation of these findings on biofilm dissolution difficult.

Several studies investigating the effect of application time on the anti‐biofilm capacity of NaOCl have yielded interesting results. It has been suggested that the time‐dependent effectiveness of NaOCl should be evaluated in relation to the bacterial species comprising the biofilm (Spratt *et al*. 2001, Bryce *et al*. 2009). Moreover, despite the tendency for enhanced anti‐biofilm capacity of NaOCl with increasing application time, it seems that biofilm maturity is a factor that could modify this observed trend (Wang *et al*. 2012, Stojicic *et al*. 2013, Du *et al*. 2014, Chau *et al*. 2015). Therefore, using clinically relevant multi‐species biofilms of standardized composition and maturity is required when the anti‐biofilm efficacy of NaOCl is investigated.

For that purpose, *Streptococcus oralis* and *Actinomyces naeslundii* were used for the development of steady‐state, dual‐species biofilms in the present study. Both bacterial species are frequently associated with persistent endodontic infections (Chávez de Paz *et al*. 2003), whilst *Actinomyces naeslundii* is also implicated in extra‐radicular infections (Siqueira 2003). Furthermore, they are known for their capacity to co‐aggregate and co‐adhere, forming robust biofilms with defined viscoelastic properties, especially when grown in a constant depth film fermenter (CDFF) (Palmer *et al*. 2003, He *et al*. 2013, Busanello *et al*. 2018). Finally, the CDFF ensures that the biofilms will reach a standardized thickness and steady‐state level of maturity (Kinniment *et al*. 1996), which are important parameters for the establishment of the biofilm model.

The volume of NaOCl applied determines the ‘reservoir’ of the available reactive NaOCl components and thereby influences the chemical potency of NaOCl and its subsequent anti‐biofilm capacity. Nevertheless, there are only a limited number of studies investigating the effect of NaOCl volume on its chemical efficacy. Increased NaOCl volume has been associated with increased dissolution of an artificial organic film from root canals submitted to a constant and continuous flow rate (Huang *et al*. 2008). On the contrary, the volume of NaOCl applied had no effect on the chemical dissolving capacity when NaOCl was delivered statically on multi‐species biofilms (Del Carpio‐Perochena *et al*. 2011). These contradictory results warrant further investigation, especially under conditions such as those met in the limited anatomical space of the root canal system, where diffusion is the principle mediator of the associated chemical events (Verhaagen *et al*. 2014).

A compromised chemical efficacy of NaOCl will result in inferior biofilm removal. The sub‐optimal anti‐biofilm effect of hypochlorite‐based formulations has been demonstrated (Norwood & Gilmour 2000, Stewart *et al*. 2001). Moreover, clinical studies showing incomplete biofilm removal following disinfection of complex root canal systems provide additional evidence on the compromised effectiveness of NaOCl to promote biofilm eradication (Nair *et al*. 2005, Ricucci & Siqueira 2010). The remaining biofilm, given the appropriate time and conditions, can re‐develop (Chávez de Paz *et al*. 2008, Shen *et al*. 2010, 2016, Ohsumi *et al*. 2015), thereby contributing to disease persistence (Siqueira & Rôças 2008). Collecting information about the architecture of the remaining biofilm could arguably aid in the development of more effective removal regimes (Peterson *et al*. 2015).

The primary objective of this study was to evaluate the influence of application time and irrigant volume on the anti‐biofilm efficacy of 2% NaOCl solution against steady‐state dual‐species biofilms of standardized thickness grown with the CDFF. Three clinical factors were taken into consideration with regard to the 2% NaOCl concentration used, namely the lack of universal consensus amongst clinicians performing root canal treatments, a cost/benefit ratio analysis of using high concentration NaOCl (lack of evidence‐based association between high NaOCl concentrations and treatment outcomes, realistic possibility of severe procedural mishaps) and the documented geographical trend towards the use of intermediate NaOCl concentrations in Europe (Slaus & Bottenberg 2002, Willershausen *et al*. 2015) in contrast to the use of high NaOCl concentrations (>5%) in the United States (Dutner *et al*. 2012, Savani *et al*. 2014). Optical coherence tomography (OCT) was used for the assessment of the anti‐biofilm efficacy of NaOCl; biofilm dissolution and disruption were the outcome measures. The secondary objective was to investigate the influence of application time and irrigant volume on the architecture of 2% NaOCl‐treated biofilms. Structural changes on the architecture of the remaining biofilms were assessed by quantifying stained biofilm components with Confocal laser scanning microscopy (CLSM) and by quantifying changes in biofilm viscoelasticity with low load compression testing (LLCT). By using a realistic contact surface area between the biofilm substrate and the 2% NaOCl solution and omitting convection, only the diffusion‐induced chemical effects were investigated.

## Materials and methods

### Biofilm formation

A CDFF was equipped with 15 sample holders. One holder included 5 saliva‐coated hydroxyapatite (HA) discs of 5 μm diameter each (Rózenbaum *et al*. 2017). The HA discs were recessed to a depth of 250 μm within the holders in order to allow the growth of biofilms of standardized thickness. For saliva coating, freeze‐dried whole saliva collected from at least 20 healthy volunteers of both genders. Saliva collection was performed in agreement with the guidelines set out by the Medical Ethical Committee at the University Medical Center Groningen, Groningen, The Netherlands (approval letter 06‐02‐2009). The lyophilized saliva was dissolved in 30 mL adhesion buffer (1.5 g L^−1^), stirred for 2 h and centrifuged at 15 000 ***g***, 10 °C for 5 min. The HA discs were exposed to the reconstituted saliva for 14 h at 4 °C under static conditions.

With regard to the bacterial composition of the biofilms used, the clinical isolates *Streptococcus oralis* J22 and *Actinomyces naeslundii* T14V‐J1 were grown as described previously (Busanello *et al*. 2018). The bacteria were streaked on blood agar plates, and a single colony was used to inoculate 10 mL modified brain heart infusion broth (37.0 g L^−1^ BHI, 1.0 g L^−1^ yeast extract, 0.02 g L^−1^ NaOH, 0.001 g L^−1^ Vitamin K1, 5 mg L^−1^ L‐cysteine‐HCl, pH 7.3) (BHI; Oxoid Ltd., Basingstoke, UK). Subsequently, *S. oralis* J22 were cultured at 37 °C for 24 h in ambient air and *A. naeslundii* T14V‐J1 were cultured at 37 °C for 48 h in an anaerobic chamber (pre‐cultures).

Pre‐cultures were used to inoculate 50 mL modified BHI (1:20 dilution) and grown for 16 h (main cultures). Bacteria were harvested by centrifugal force (6350 ***g***) and washed twice in sterile adhesion buffer (0.147 g L^−1^ CaCl_2_, 0.174 g L^−1^ K_2_HPO_4_, 0.136 g L^−1^ KH_2_PO_4_, 3.728 g L^−1^ KCl in sterile demineralized water, pH 6.8). The bacterial pellets were suspended in 10 mL sterile adhesion buffer and sonicated intermittently in ice water for 3 × 10 s at 30 W (Vibra cell model 375, Sonics and Materials Inc., Newtown, CT, USA) to break bacterial chains. Bacteria were counted in a Bürker‐Türk counting chamber (Marienfeld‐Superior, Lauda‐Königshofen, Germany) to determine the concentration. The mono‐suspensions were diluted in sterile adhesion buffer to prepare a dual‐species bacterial suspension of a concentration of 6 × 10^8^ bacteria mL^−1^ for *S. oralis* J22 and 2 × 10^8^ bacteria mL^−1^ for *A. naeslundii* T14V‐J1. Following, 100 mL of the suspension was introduced dropwise in the CDFF over 1 h, whilst the CDFF table with the holders was kept in constant slow rotation. Subsequently, the rotation was stopped for 30 min to allow for the bacteria to adhere to the HA substrate. Finally, rotation was resumed, and the biofilms were grown for 96 h at 37 °C under continuous supply of modified BHI with a rate of 45 mL h^−1^.

### Static application of 2% NaOCl using different volumes and for different time intervals

The biofilms were challenged with static application of 2% NaOCl (Sigma‐Aldrich, St Louis, MO, USA) in order to evaluate only the diffusion‐induced chemical effect. Volumes of 20‐ or 40 μL were gently pipetted over the biofilm samples and left undisturbed for 60‐, 120‐ or 300 s. To ensure the proper concentration of the NaOCl, a thiosulfate titration assay was performed before every experiment. After treatment application, NaOCl was neutralized by gently pipetting 4.23% sodium thiosulfate solution (Na_2_S_2_O_3_, Sigma‐Aldrich) over the biofilm samples.

### Optical coherence tomography

Biofilm evaluation with OCT was carried out before and after treatment with 2% NaOCl. The biofilms were kept in a volumetric jar with adhesion buffer. Real‐time 2D cross‐sections of the biofilm were acquired with an OCT scanner (Thorlabs, Newton, NJ, USA) using a field of view (FOV) size of 45 mm, refraction index of 1.33, and processed with ThorImage OCT software (Thorlabs).

**Figure 1:**
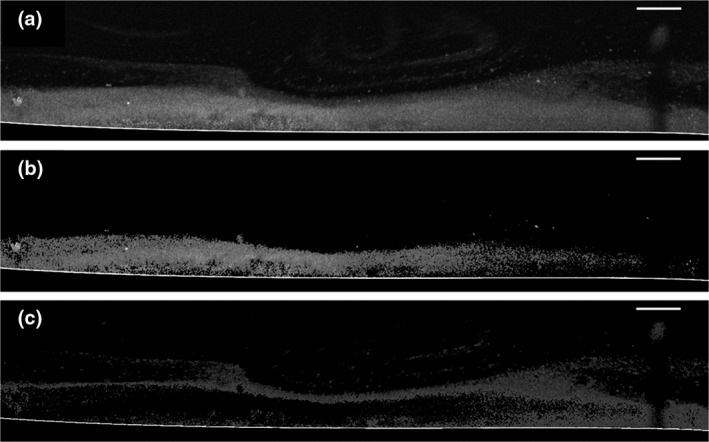
Multilevel greyscale thresholding from a representative 2% NaOCl‐treated CDFF biofilm. Identification of different biofilm layers imaged with the OCT. The degree of coherence of each layer was correlated to its corresponding greyscale level. (a) Original image of biofilm acquired with OCT, split after multilevel thresholding in (b) coherent layer (higher greyscale level pixel intensity) and (c) disrupted layer (lower greyscale level pixel intensity) (scale bar: 250 μm).

To increase the reproducibility of the image analysis, ImageJ (Fiji) was used to calculate the distance in every column of pixels between the substrate and top of the biofilm (4500 rows of pixels). To improve the accuracy of the data, an image analysis to manage different thresholds in one image was selected (Otsu 1979, Liao *et al*. 2001). This resulted in the identification of different layers in the biofilm. The layer exhibiting the lower greyscale pixel intensity became easily detached from the underlying biofilm just by passing the biofilm through an air‐liquid interface and was assigned to the term ‘disrupted layer’. The layer with the higher greyscale pixel intensity remained relatively undisturbed whilst attached to the substrate and was assigned to the term ‘coherent layer’ (Fig. 1). Biofilm dissolution and biofilm disruption were chosen as outcome measures. For biofilm dissolution, the per cent reduction of the coherent layer (thereof also called, per cent biofilm reduction) was calculated based on the pre‐ and post‐treatment OCT height measurements of the coherent layer. For biofilm disruption, the per cent increase of the disrupted layer was calculated based on the pre‐ and post‐treatment OCT height measurements of the disrupted biofilm layer.

### Confocal laser scanning microscopy (CLSM)

Biofilms were stained with live/dead stain (BacLight™; Invitrogen, Breda, The Netherlands) in a ratio of 1:3 for 20 min and with calcofluor white to stain the EPS (20 μL mL^−1^, 3.8 mmol L^−1^) for 10 min. After removal of the staining, biofilms were submerged in 15 mL adhesion buffer and kept protected from light until imaging. A confocal laser microscope (Leica TCSSP2; Leica Microsystems GmbH, Heidelberg, Germany) was used to record a stack of images from two different randomly selected locations on each biofilm with an 8 × 40 mm water objective lens, with 1024 × 1024 pixels. Image analysis was performed with the COMSTAT software, and the ratio of red (dead bacteria), green (live bacteria) and blue (EPS) to the total biovolume was calculated (Heydorn *et al*. 2000). This was expressed as the relative percentage of each stained component (live bacteria: green, dead bacteria: red, EPS: blue) to the total biomass (total bacteria and EPS).

### Low load compression testing (LLCT)

**Figure 2:**
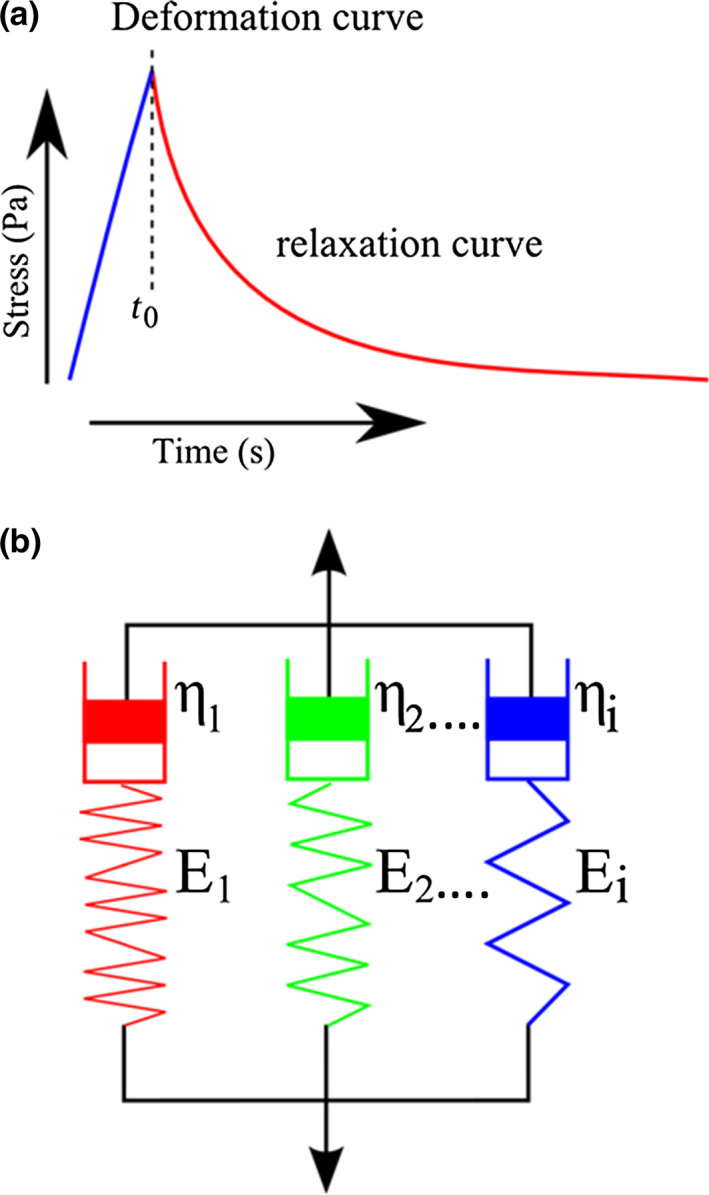
Representation of viscoelastic model for biofilms (modified from He *et al*. 2013). (a) Deformation curve consisting of applied stress (Pa) until t_0_ and relaxation over time (s). (b) Schematic presentation of the generalized Maxwell model, comprised of spring constant E_i_, viscosity ƞ_i_.

The viscoelastic properties of the biofilms were determined by performing stress relaxation measurements on the low load compression tester (Sharma *et al*. 2011, He *et al*. 2013, Peterson *et al*. 2013, Busanello *et al*. 2018). The biofilms were compressed to a deformation of 20% in 1 s which was then held constant for 100 s. The relaxation was monitored over time and normalized over the cross‐sectional area of the plunger to calculate the induced stress. The percentage change in induced stress occurring within 100 s from its initial value was termed the percentage stress relaxation (R). Measured relaxation curves for each biofilm were modelled using a generalized Maxwell model. E(t) represents the total stress exerted by the biofilm, which decreases with time, divided by the imposed constant strain of 0.2. Measured E(t) is modelled as the sum of four Maxwell elements, with a spring constant *E*_i_, and characteristic relaxation time constant, τ_i_ (Fig. 2). The relative importance of each element was expressed as the percentage of its spring constant to the sum of the spring constants of all elements at 0 s, that is before relaxation starts. Allocating each Maxwell element, based on its relaxation time constant, to a specific biofilm component allowed for quantification of the contribution of each component to the overall biofilm viscoelasticity (Busanello *et al*. 2018). Samples were kept submerged in buffer during measurements, and due to the sensitivity of the weight and the duration of the measurements (100 s), a correction for water evaporation was applied.

### Statistical analysis

Statistical analysis was carried out using SPSS software (version 23.0; IBM Corp., Armonk, New York, USA). A two‐way analysis of variance (2‐way anova) was performed. ‘Time’ and ‘Volume’ were the two independent variables. Tukey's HSD *post hoc* tests were performed to analyse the effect of ‘Time’ (3‐level independent variable) on the outcome measures. Further analysis of the interaction between the two independent variables (‘Time’ x ‘Volume’) was carried out through simple effect analysis (SPPS Syntax). Data are expressed as mean and standard deviation (SD). The level of statistical significance was set at a <0.05.

## Results

### Anti‐biofilm efficacy of 2% NaOCl

#### Biofilm dissolution (per cent reduction of coherent layer)

**Figure 3:**
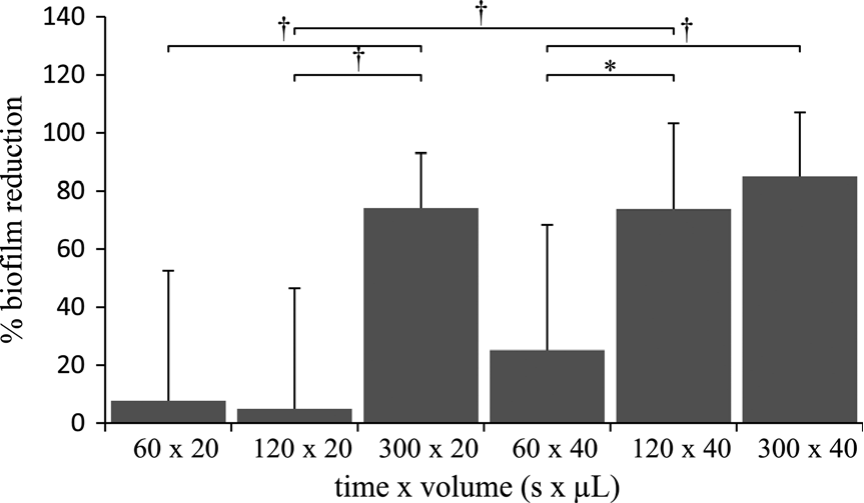
Time‐ and volume‐dependent biofilm dissolution upon statical exposure of CDFF biofilms (limited surface contact area) to 2% NaOCl. Percentage biofilm reduction (as expressed through the % decrease biofilm coherent layer) is presented and compared across all levels of the two independent variables (‘Time’ x ‘Volume’). Values are presented as mean and standard deviation (SD). Statistical significance is indicated by * for *P *≤ 0.05 and † for *P *≤ 0.01.

The main effect of ‘Time’ was statistically significant (*P *= 0.001), meaning that when ‘Volume’ was not taken into account in the 2‐way anova, the results revealed that increasing time resulted in the reduction of the coherent biofilm layer; 300 s exposure to 2% NaOCl led to significantly more biofilm removal compared to 60 s (*P *< 0.001) and 120 s (*P *= 0.003) (Table 1). The main effect of ‘Volume’ was statistically significant (*P *= 0.008), meaning that when ‘Time’ was not taken into account in the 2‐way anova, the results revealed that 40 μL resulted in significantly more biofilm removal compared to the 20 μL (Table 1). The interaction between ‘Time’ and ‘Volume’ reached an almost significant value in the 2‐way anova (*P *= 0.075), and therefore, simple effect analysis was considered necessary for exploring the influence of each level from the two independent variables on biofilm dissolution. According to this, upon application of a smaller volume (20 μL), no significant difference in biofilm removal was observed between the low and intermediate application times (60‐ and 120 s), but the biofilm coherent layer was significantly reduced when time increased (300 s) (*P *< 0.01). Upon higher volume application (40 μL), no significant difference in biofilm removal was recorded between the intermediate and higher application times (120‐ and 300 s), but both exposure times induced a significant biofilm reduction compared to the low exposure time (60 s) (*P *< 0.05). In the low and high exposure times (60‐ and 300 s), changes in the volume of 2% NaOCl applied did not lead to significant differences in biofilm removal. However, in the intermediate exposure time (120 s), the coherent biofilm layer was significantly reduced when the biofilm was exposed to 40 μL compared to 20 μL 2% NaOCl (*P *= 0.001) (Fig. 3).

**Table 1:** Mean and standard deviation (SD) of biofilm dissolution (per cent reduction coherent layer) and biofilm disruption (per cent increase disrupted layer) after static exposure of CDFF biofilms to 2% NaOCl for variable time periods and application volumes

	s	% reduction coherent layer			% increase disrupted layer			s	
		Mean	SD	*P*‐value	Mean	SD	*P*‐value		
‘Time’ (*P *= 0.001)*	60	16.5	41.8	<0.001**	25.3	24.1	<0.001**	60	‘Time’ (*P *= 0.001)*
	120	34.5	46.4	0.003**	44.8	33.7	0.015**	120	
	300	81.3	24.0	–	72.6	25.3	–	300	

	μL	% reduction coherent layer			% increase disrupted layer			μL	
		Mean	SD	*P*‐value	Mean	SD	*P*‐value		
‘Volume’ (*P *= 0.008)*	20	22.1	41.4	–	30.2	27.0	–	20	‘Volume’ (*P *= 0.002)*
	40	65.7	41.5	0.008***	65.1	30.8	0.002***	40	

* Significant difference yielded from main effect analysis of each independent variable (two‐way anova).

** Significant difference when compared to 300 s (Tukey's HSD *post hoc* test).

*** Significant difference when compared to 20 μL (two‐way anova).

#### Biofilm disruption (per cent increase of disrupted layer)

The main effect of ‘Time’ was significant (*P *= 0.001), meaning that when ‘Volume’ was not taken into account in the 2‐way anova, the results showed that increasing time resulted in increasing biofilm disrupted layer; 300 s exposure to 2% NaOCl led to significantly more biofilm disruption compared to 60 s (*P *< 0.001) and 120 s (*P *= 0.015) (Table 1). The main effect of ‘Volume’ was significant (two‐way anova,* P *= 0.002), meaning that when ‘Time’ was not taken into account in the 2‐way anova, the results revealed that the greater volume of 40 μL 2% NaOCl resulted in significantly more biofilm disruption compared to the lower volume of 20 μL (Table 1). No significant interaction between ‘Time’ and ‘Volume’ was noted (*P *= 0.143), and thereby no further simple effect analysis was performed.

### Changes in the architecture of the remaining biofilms

#### Confocal laser scanning microscopy

**Figure 4:**
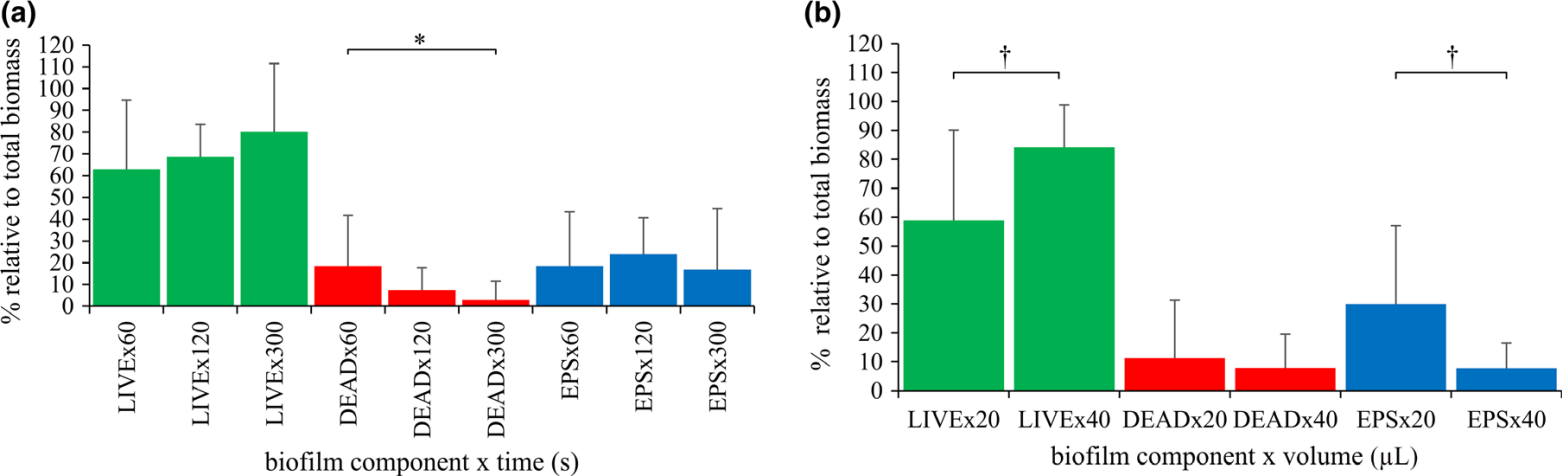
Confocal laser scanning microscopy (CLSM) quantification of stained biofilm components. Time‐ and volume‐dependent changes in the amount of live and dead bacteria, and extracellular polymeric substances (EPS) relative to the total biomass upon statical exposure of CDFF biofilms (limited surface contact area) to 2% NaOCl. Two‐way anova main effects of (a) ‘Time’ and (b) ‘Volume’, on percentage relative amount of stained biofilm components (green: live bacteria, red: dead bacteria, blue: EPS) to the total biomass. Values are presented as mean and standard deviation (SD). Statistical significance is indicated by * for *P *≤ 0.05 and † for *P *≤ 0.01.

**Figure 5:**
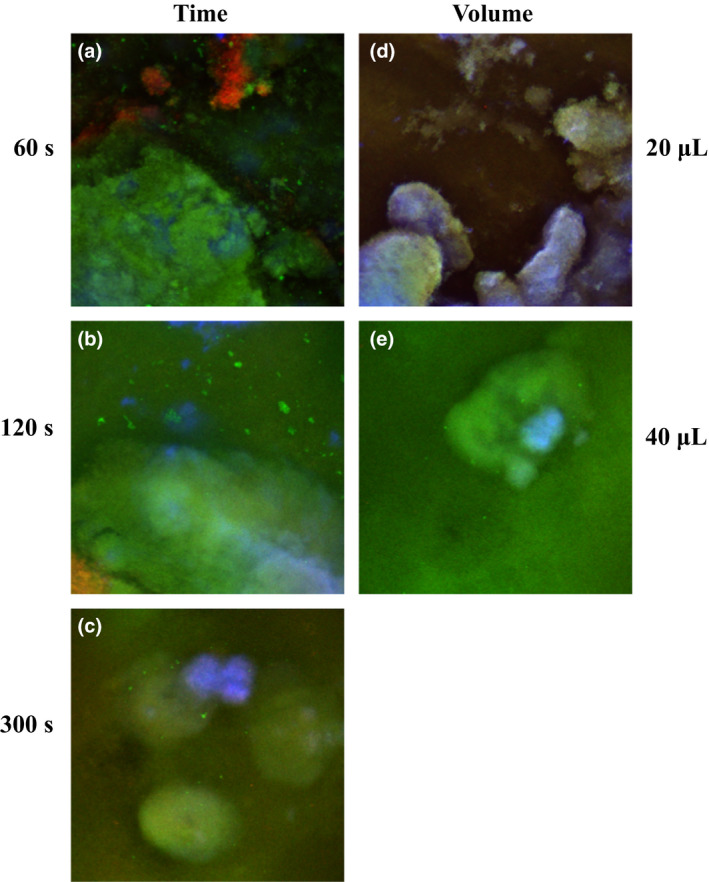
Representative CLSM overview micrographs after static application of 2% NaOCl solution on CDFF biofilms for different time periods and of different volumes. (a‐c) CLSM micrographs from the different exposure times. A considerably high presence of dead bacteria (red stain) is visualized after 60 s treatment with 2% NaOCl (a), whereas almost complete absence of dead bacteria and possible biofilm ‘highly resistant spots’ persisting even after prolonged treatment are visualized after 300 s treatment (c). No remarkable differences are visualized after 2% NaOCl treatment in the amount of EPS material (blue stain) and live bacteria (green stain) amongst the three ‘Time’ groups. (d, e) CLSM micrographs from the different application volumes. A considerably lower presence of live bacteria (green stain) and higher presence of EPS material (blue stain) are visualized after application of 20 μL of 2% NaOCl (d) compared to the 40 μL (e), where considerably more live bacteria (green) are also observed.

‘Time’ x ‘Volume’ interaction did not yield any statistical significance, thereby leaving only the main effects of ‘Time’ and ‘Volume’ separately for further interpretation. The main effect of ‘Time’ reached significance for the dead bacteria (*P *= 0.05) in the 2‐way anova. As time was increasing, less dead bacteria were present, with the highest exposure time (300 s) resulting in significantly less dead bacteria within the biomass compared to lowest exposure time (60 s) (Fig. 4a). The main effect of ‘Volume’ reached statistical significance for the live bacteria (*P *= 0.004) and EPS (*P *= 0.003) in the 2‐way anova. Forty microlitres of 2% NaOCl resulted in significantly greater percentage live bacteria, compared to the 20 μL (*P *= 0.004). Also, 40 μL resulted in significantly less percentage EPS within the remaining biomass compared to the 20 μL (*P *= 0.003) (Fig. 4b). Representative images are shown in Fig. 5.

#### Low load compression testing

**Figure 6:**
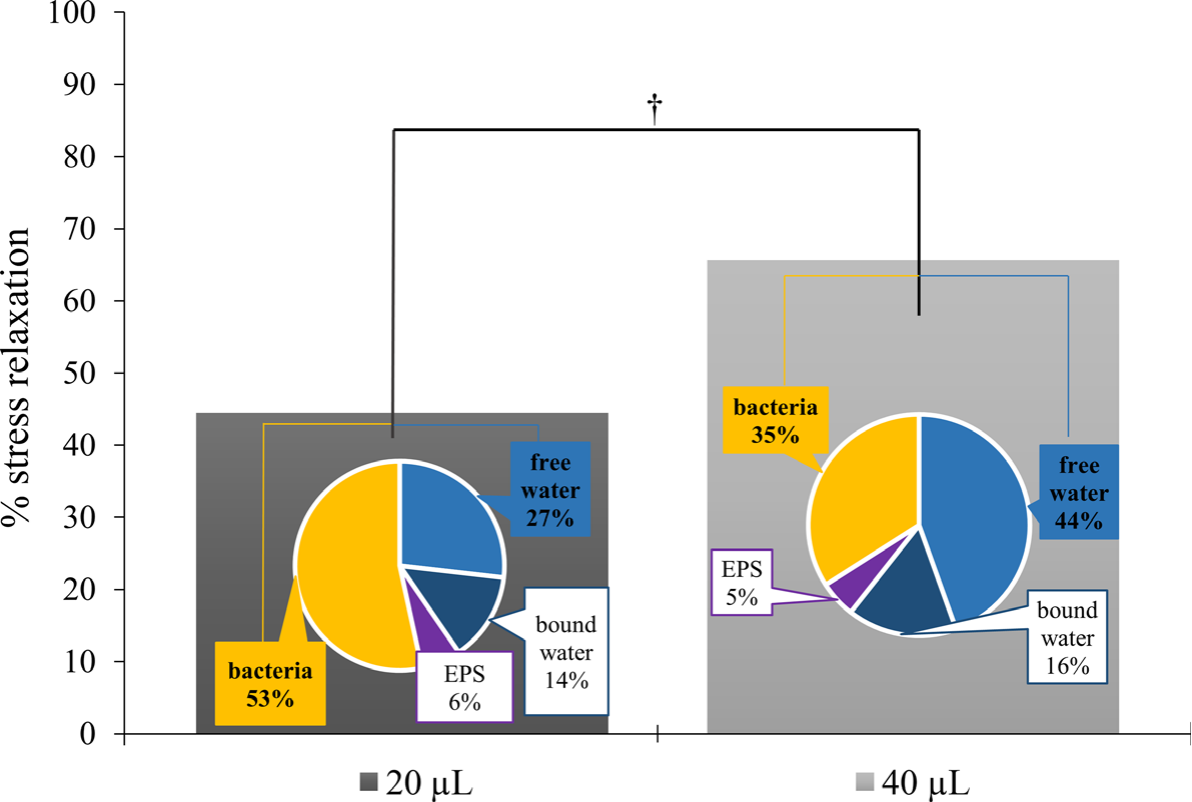
Viscoelastic analysis of CDFF biofilms after exposure to different volumes of 2% NaOCl. The bar graph shows the effect of NaOCl volume (20 and 40 μL) on percentage biofilm stress relaxation after the 20% instantaneous constant deformation of the NaOCl‐treated biofilms (remaining biofilms). Statistical significance is indicated by † for *P *≤ 0.01. The pie charts show the results from the stress relaxation curve fitting analysis with a generalized Maxwell viscoelasticity model. Analysis yielded differences between specific viscoelastic elements when different volumes of 2% NaOCl were applied (20 and 40 μL). By allocating each viscoelastic element (E_1_, E_2,_ E_3_ and E_4_) to respective biofilm components (free water, bound water, extracellular polymeric substances‐EPS and bacteria), the percentage contribution of each component on the overall viscoelastic behaviour of the remaining biofilms was calculated. The contribution of bacteria (depicted in orange colour) and free water (depicted in light blue colour) differed statistically significant for the two different volumes. Statistical significance is indicated by † for *P *≤ 0.01.

Based on previous findings, each Maxwell element was allocated to a specific biofilm structural component. Accordingly, E_1_ (τ_1_ < 0.5 s) was associated with free water, E_2_ (0.5 < τ_2_ < 3 s) with bound water, E_3_ (3 < τ_3_ < 100 s) with EPS and E_4_ (100 s < τ_4_) with bacteria (Busanello *et al*. 2018). The effects of ‘Time’, ‘Volume’ and ‘Time x Volume’ on the percentage stress relaxation and on the percentage contribution of each biofilm component to the overall biofilm viscoelasticity were assessed. ‘Time x Volume’ interaction did not yield any statistical significance. Also, the main effect of ‘Time’ did not yield any statistical significance, thereby leaving only the main effect of ‘Volume’ for further interpretation. The main effect of ‘Volume’ was statistically significant for the stress relaxation (*P *= 0.003), for the relative importance of free water (*P *= 0.01) and bacteria (*P *= 0.008) (Fig. 6). Forty microlitres of 2% NaOCl resulted in significantly higher stress relaxation (mean = 64.3, SD = 16.2) compared to the 20 μL (mean = 44.5, SD = 20.6) (*P *= 0.003). Also, 40 μL resulted in a significantly higher relative importance of free water (mean = 44.2, SD = 18.2) (*P *= 0.01) and a significantly lower importance of bacteria (mean = 35.2, SD = 16.3) (*P *= 0.008) compared to the 20 μL (mean = 26.8, SD = 16.3 for free water and mean = 53.4, SD = 21.2 for bacteria) (Fig. 6). The importance of EPS and bound water remained more or less unaffected.

## Discussion

Generally, *in vitro* models often show complete biofilm dissolution after static application of NaOCl over biofilm samples. Interestingly, this outstanding anti‐biofilm capacity of NaOCl is not replicable in clinical practice (Nair *et al*. 2005, Ricucci & Siqueira 2010), even though the augmenting effect of irrigant flow during irrigation would be expected to enhance biofilm removal. The physical constraints posed by the intricate root canal anatomy and the modifying effect of the dentine substrate on the potency of root canal medicaments are held accountable for this discrepancy (Haapasalo *et al*. 2000). However, methodological‐ and biofilm‐related factors may also contribute to the lack of consistency between the *in vitro* and *in vivo* effectiveness of NaOCl.

The anti‐biofilm efficacy of NaOCl is mostly tested against single‐species biofilms of different age and unknown structure. The samples are often grown on various substrates and under conditions which bear only a minor resemblance to the clinical setting (Haapasalo & Shen 2012). However, biofilm growth substrate (Chávez de Paz *et al*. 2010), biofilm age (Chau *et al*. 2015), biofilm species composition (Bryce *et al*. 2009) and biofilm structure (Busanello *et al*. 2018) are all factors that influence the response of biofilms to the applied biocides. In particular, a recent study has demonstrated that steady‐state, dual‐species, gram‐positive biofilms, grown on saliva‐coated hydroxyapatite discs, showed dense bacterial presence and low content in extracellular polymeric substances (EPS) and were less prone in 2% NaOCl‐mediated dissolution (Busanello *et al*. 2018). Based on these findings, similar biofilms were developed in the present study in order to test factors affecting the chemical efficacy of 2% NaOCl.

From a methodological standpoint, it has to be noted that saliva lyophilization does not guarantee sterilization. However, prior to freeze‐drying, the saliva was centrifuged twice to remove any micro‐sized debris, including bacterial cells. This ensures a considerably decreased remaining bacterial load. After salivary protein adsorption, the surface of the HA discs was inoculated with a large number of *S. oralis* and *A. naeslundii* bacterial cells, which are eventually expected to overwhelm any remaining saliva‐derived bacterial cells present. Therefore, no growth of and interference from extraneous bacterial species have been observed in these dual‐species CDFF biofilms (He *et al*. 2013, Busanello *et al*. 2018). Finally, the importance of saliva conditioning in the co‐adhesion of the particular bacterial species used in this study has been already demonstrated (Bos *et al*. 1996).

The concept of limited surface contact was also taken into consideration. In the majority of relevant studies, biofilm samples are fully immersed in an excess of a given biocide. This deviates considerably from the actual irrigant application. Within the confined space of the root canal, only limited contact between a small volume of NaOCl and the biofilm takes place. This study was set up to investigate the diffusion‐driven chemical efficiency and efficacy of 2% NaOCl. Therefore, the limitations related to the ‘one‐off’ NaOCl application and the lack of irrigant flow should be acknowledged. This static mode of NaOCl application does not allow for any added benefits from the repeated irrigant supply and convection to build up and consequently be investigated. However, these limitations could be circumvented in future studies with the use of OCT, as multiple assessments on the same biofilm sample (Wagner & Horn 2017) and ‘real‐time’ evaluation flow cell systems are feasible (Rasmussen *et al*. 2016). Overall, this study has accounted for methodological‐ and biofilm‐related considerations in an attempt to standardize the *in vitro* conditions and test the chemical efficacy of 2% NaOCl under potentially harsh clinical conditions.

The application of 2% NaOCl resulted in a dual action on the tested biofilms, as this was revealed by the OCT. In agreement with the notion that a single outcome measure may be insufficient to demonstrate the action of a given biocide against biofilms (Bryce *et al*. 2009), two outcome measures for biofilm evaluation were used in this study. This was based on the clear distinction between a coherent and disrupted biofilm layer (Busanello *et al*. 2018), thus enabling the quantification of biofilm dissolution and disruption. Although biofilm disruption and dissolution upon exposure of the biofilms to chemical solutions take place simultaneously, these two processes should be examined separately. Biofilm disruption arguably represents an intermediate stage before biofilm dissolution occurs, which leads to an easily detachable superficial biofilm layer. Visualization and subsequent quantification of the disrupted biofilm layer make it possible to evaluate a chemical effect other than dissolution, which may reflect the structural alterations that the biofilm undergoes after the application of a potent reactant such as NaOCl.

By increasing the time of exposure, the disrupted layer showed a tendency to increase. Interestingly, no significant change was detected between the low and intermediate time intervals (60 and 120 s), whilst significant disruption was noted with a considerable increase in the exposure time (300 s). This indicates that 2% NaOCl shows a progressive and time‐dependent reaction with the underlying organic substrate that becomes substantial only after a given time interval is surpassed. Whether this effect ‘plateaus’ after a specific period of time warrants further investigation.

An increase in the applied volume caused a significant increase in biofilm disruption (a two‐fold volume increase resulted in an almost two‐fold biofilm disruption). Although further investigation is again needed to reveal whether a ‘volume plateau’ exists, this finding clearly shows how volume affects the chemical efficacy of 2% NaOCl when tested against a limited and standardized surface area. From a clinical point of view, this highlights the importance of providing the root canal system with a larger ‘NaOCl reservoir’ in order to enhance the reactivity of the specific irrigant when it comes in limited contact with the underlying biofilm. With regard to the clinical significance of the disrupted layer, this has yet to be elucidated.

The biological significance of the passive ‘biofilm dispersal’ (Kaplan 2010) that possibly occurs due to the detachable nature of this layer is currently unknown. Passing the biofilms through an air–liquid interface is sufficient to induce detachment of the superficial chemically affected biofilm layer. This implies that subsequent physical shear forces developed through convection currents (irrigant flow) should be adequate to remove it completely. However, the possibilities that disrupted biofilm residues that are not adequately removed adhere to another surface and re‐colonize areas of the root canal or re‐cohere to any remaining biofilm cannot be excluded. Arguably, this hinders the task of biofilm elimination and allows for biofilm re‐development, with a potentially adverse effect on the resolution of periapical disease.

Acknowledging biofilm dissolution as the primary aim of root canal disinfection, investigating the factors that mediate this event is of utmost importance. In line with the previous results regarding the impact of time and volume on biofilm disruption, the main effects of these two parameters on biofilm dissolution (coherent biofilm layer) followed the same trend. However, a further analysis of the interaction between these two independent variables yielded findings with potential clinical relevance.

Upon application of a smaller volume of 2% NaOCl (20 μL), significant biofilm removal was observed only in the maximum exposure time (300 s). However, when a larger volume was applied (40 μL), then significant biofilm dissolution was recorded at a relatively shorter exposure time (120 s). Eventually, this difference ceased to exist when the maximum exposure time was reached. This shows that a fine balance between irrigation time and volume exists, as by simply increasing the applied volume of 2% NaOCl, greater biofilm removal can be achieved in less time. As a clinical consequence of this compensating equilibrium, prolonged exposure of dentine to this strong oxidative agent could be avoided, provided that an ample volume of irrigant is available for root canal disinfection. In this manner, significant biofilm removal can be achieved and adverse effects on the physico‐mechanical properties of dentine minimized (Pascon *et al*. 2009).

Although speculative in nature, the following hypothesis could account for this finding. A larger NaOCl ‘reservoir’ (higher NaOCl volume) provides the reacting NaOCl‐biofilm system with a greater availability of NaOCl reactant. By increasing the net amount of reactive NaOCl molecules that come into contact with a defined biofilm surface area, an increase in the diffusion‐driven transport of NaOCl molecules into the biofilm is expected. As a result, deeper biofilm layers are affected and transit to a disrupted state, thus becoming more susceptible to removal. Arguably, a similar increase in the net amount of reactive NaOCl can be achieved by employing NaOCl of higher concentration (>2%). Indeed, higher concentrations of NaOCl are often used clinically, although the additional benefit of its use on the treatment outcome has yet to be established. Higher concentrations of NaOCl demonstrate good anti‐biofilm efficacy *in vitro* (Clegg *et al*. 2006), but cause significant alterations to the physico‐chemical properties of dentine as well (Marending *et al*. 2007). Further research is warranted that would take into account changes occurring both at the biofilm and dentine substrate after exposure to NaOCl of variable concentration, time, and volume gradients.

Summarizing the above, it could be argued that depending on the volume applied, the NaOCl reactant is associated with a different pattern of escalating reactivity with the biofilm as time progresses (‘*volume‐dependent peak time threshold’* of NaOCl chemical efficacy). Although eventually (300 s) the same final outcome is reached regardless of the applied volume, adding more volume of NaOCl reactant on a limited organic surface area seems to accelerate one of the aftermaths of NaOCl reactivity, namely biofilm dissolution.

With regard to the architectural composition of the biofilms that remained after the ‘one‐off’ chemical attack, only the ‘Volume’ variable yielded significant changes on the viscoelastic properties of 2% NaOCl‐treated biofilms. First, the recorded stress relaxation of the remaining biofilms that were treated with a higher volume of 2% NaOCl was considerably higher compared to the lower volumes. From a physical standpoint, this means that any developed stress within these biofilm structures can be effectively relieved. Practically, mechanical forces can dissipate more easily within this chemically affected biofilms, thus reducing the likelihood of structural failure and uncontrolled detachment that are expected to occur with the physical shear force development during irrigant flow (Rupp *et al*. 2005).

Secondly, a more detailed analysis of the contribution of the different elements on the overall viscoelastic properties of the biofilms revealed again a significant role for the variable ‘Volume’. More specifically, by increasing 2% NaOCl volume, a significant increase in the contribution of the free water and a significant decrease in the contribution of the bacteria on the viscoelasticity of biofilms were recorded. A decreased water contribution and an increased bacterial contribution have been linked to increased penetration of chlorhexidine within similarly grown CDFF biofilms (He *et al*. 2013). This occurs due to the diminution of the dilution effect on the penetrating anti‐biofilm solution (decreased involvement of the water element) and the extensive bacterial re‐arrangement that results in a biofilm structure allowing for deeper chlorhexidine penetration (increased involvement of the bacterial element). By extrapolation, the present findings suggest that when larger volumes of 2% NaOCl are applied the architectural biofilm composition shifts in such a way that makes the biofilm less susceptible to chlorhexidine penetration. Thus, any subsequent use of chlorhexidine, as this is proposed by disinfection regimes where a final rinse with CHX is advocated (Zehnder 2006, Basrani & Haapasalo 2012), is not expected to exert any significant anti‐biofilm action due to limited penetration in the biofilm residues. In combination with recent evidence associating the use of chlorhexidine with biofilm stiffening and contraction, inadequate biofilm removal, (Hope & Wilson 2004, Brindle *et al*. 2011, Shen *et al*. 2016, Busanello *et al*. 2018) and high cytotoxic effects when combined with NaOCl (Nocca *et al*. 2017), the notion about revisiting the need for chlorhexidine as an adjunct in root canal disinfection is further supported (Busanello *et al*. 2018).

Quantification of the stained biofilm components from the CLSM‐acquired images showed that prolonged exposure of CDFF biofilms to 2% NaOCl resulted in a significant decrease in the relative amount of dead bacteria, without any further differences detected in the amount of live bacteria and EPS amongst the time groups. This counter‐intuitive finding supports the assumption that 2% NaOCl has a stronger effect on bacterial cells that already exhibit defects on their cell membrane, thereby rendering them more prone to removal as exposure time advances.

With regard to the volume, increasing the 2% NaOCl reservoir over the biofilm samples led to a significant decrease in the amount of EPS and a respective significant increase of the live bacteria. The EPS reduction is related to the proteolytic and saccharolytic properties of NaOCl (Naenni *et al*. 2004, Urano & Fukuzaki 2005, Tawakoli *et al*. 2015) that result in the decomposition of the structural backbone of the biofilm matrix. As far as the increased bacterial viability is concerned, the gradual development of the biofilm in the CDFF could account for this finding. More specifically, after the biofilms reach their pre‐determined thickness, the continuous compaction exerted by the scrapers results in a specific bacterial stratification, with more dead cells in the superficial layer and more live cells beneath (Hope & Wilson 2006, He *et al*. 2013, Busanello *et al*. 2018). Taking into consideration the increased biofilm disruption and dissolution demonstrated in the present study when higher 2% NaOCl volume was applied, it seems logical that the upper layers containing mostly dead bacteria are removed first. As a consequence, the immediate underlying biofilm layer containing more live bacteria is directly submitted to CLSM imaging. In addition, through the decrease of the ‘blue signal’ associated with the EPS reduction, the ‘green signal’ (live bacteria) stands out. Lastly, the inherent shortcomings of the dead/live staining need to be taken into consideration, namely, with the staining penetration depth being around 60 μm, only the superficial residual biofilm layers are subjected to evaluation. This leaves the deeper biofilm strata where more dead bacteria are anticipated out from the quantification process.

## Conclusions

This study investigated the influence of two irrigation variables, namely exposure time and volume application, on the chemical anti‐biofilm capacity of 2% NaOCl solution. Biofilm disruption and dissolution were identified as different outcome parameters and accordingly examined during the evaluation of the diffusion‐dependent chemical efficacy of 2% NaOCl. The overall results demonstrated that by increasing exposure time and irrigant volume the chemical anti‐biofilm capacity is enhanced. However, it was noted that by increasing 2% NaOCl volume, significant biofilm dissolution could be achieved in less time. A fine‐tuning between time and volume could aid in devising NaOCl‐based effective biofilm disinfection clinical strategies that would induce less damage to the underlying dentine. Finally, the time‐ and volume‐dependent alterations of the architecture of the remaining biofilms were examined through CLSM imaging and LLCT for biofilm viscoelasticity. Via CLSM, the EPS‐lytic action of 2% NaOCl was corroborated, whilst some inherent flaws associated with its use were highlighted. Via LLCT, the viscoelasticity profile of the remaining biofilms was studied. Based on the current observations and combined with previous findings, the anti‐biofilm effectiveness of chlorhexidine‐based supplemented irrigation regimes was challenged. Defining and standardising methodological parameters concerning biofilm growth and irrigant application is critical in order to obtain clinically meaningful results from *in vitro* studies that examine the anti‐biofilm capacity of various biocides. Also, employing ‘noninvasive’ methods that are less prone to methodological and evaluation bias improves outcome assessment.

## Conflict of interest

All other authors state explicitly that there are no conflicts of interest in connection with this article.
